# Identification of a novel susceptibility locus for juvenile idiopathic arthritis by genome-wide association analysis

**DOI:** 10.1002/art.24179

**Published:** 2009-01

**Authors:** Anne Hinks, Anne Barton, Neil Shephard, Steve Eyre, John Bowes, Michele Cargill, Eric Wang, Xiayi Ke, Giulia C Kennedy, Sally John, Jane Worthington, Wendy Thomson, British Society of Paediatric and Adolescent Rheumatology Study Group

**Affiliations:** 1 University of ManchesterManchester, UK; 2 University of SheffieldSheffield, UK; 3 AffymetrixSanta Clara, California; 4 PfizerKent, UK

## Abstract

**Objective:**

Juvenile idiopathic arthritis (JIA) is a chronic rheumatic disease of childhood. Two well-established genetic factors known to contribute to JIA susceptibility, *HLA* and *PTPN22*, account for less than half of the genetic susceptibility to disease; therefore, additional genetic factors have yet to be identified. The purpose of this study was to perform a systematic search of the genome to identify novel susceptibility loci for JIA.

**Methods:**

A genome-wide association study using Affymetrix GeneChip 100K arrays was performed in a discovery cohort (279 cases and 184 controls). Single-nucleotide polymorphisms (SNPs) showing the most significant differences between cases and controls were then genotyped in a validation sample of cases (n = 321) and controls, combined with control data from the 1958 UK birth cohort (n = 2,024). In one region in which association was confirmed, fine-mapping was performed (654 cases and 1,847 controls).

**Results:**

Of the 112 SNPs that were significantly associated with JIA in the discovery cohort, 6 SNPs were associated with JIA in the independent validation cohort. The most strongly associated SNP mapped to the *HLA* region, while the second strongest association was with a SNP within the *VTCN1* gene. Fine-mapping of that gene was performed, and 10 SNPs were found to be associated with JIA.

**Conclusion:**

This study is the first to successfully apply a SNP-based genome-wide association approach to the investigation of JIA. The replicated association with markers in the *VTCN1* gene defined an additional susceptibility locus for JIA and implicates a novel pathway in the pathogenesis of this chronic disease of childhood.

Juvenile idiopathic arthritis (JIA) is a generic term for arthritis that persists for more than 6 weeks and has an onset before the age of 16 years. It is the most common chronic rheumatic disease of childhood, with a prevalence of 1 in 1,000 persons. The clinical phenotype of JIA is complex. In the current International League of Associations for Rheumatology (ILAR) classification system, the disease is divided into 7 clinical subgroups, and outcome varies according to phenotype (1).

The cause of JIA is complex, involving both genetic and environmental risk factors. To date, only 2 genetic risk factors, *HLA* and the *PTPN22* gene, have been unequivocally confirmed as JIA susceptibility genes in multiple populations. Other genes, such as *MIF*, *IL6*, *IL10*, and *TNF*, show some evidence of association with JIA in different populations and subtypes (2). However, it has been hypothesized that these genes account for only a small proportion of the total genetic contribution to disease, and there are likely to be other susceptibility loci, the identification of which may lead the way to a greater understanding of the pathways involved in the disease pathogenesis and, ultimately, to new therapies.

The recent trend for taking a “hypothesis-free” approach with the use of genome-wide association studies has proved to be highly successful in identifying genetic risk factors for other complex diseases, such as rheumatoid arthritis and type 2 diabetes mellitus (3). We therefore chose to adopt this strategy to identify novel JIA susceptibility loci. We performed a multistage case–control association study investigating 112,496 single-nucleotide polymorphisms (SNPs) spanning the genome in a discovery sample set of JIA cases and controls, followed by validation of significantly associated polymorphisms in an independent sample set. Fine-mapping of one of the replicated regions implicated the *VTCN1* gene in JIA susceptibility.

## PATIENTS AND METHODS

### Study overview

A multistage case–control association study was undertaken. In the first stage, genotype frequencies in a subset of JIA cases were compared with those in population controls (discovery cohort), using an Affymetrix GeneChip 100K array (Affymetrix, Santa Clara, CA). SNPs showing evidence of association in that dataset (*P* ≤ 0.001) were genotyped in the remaining JIA cases (validation cases) and in an independent cohort of population controls. Genotype frequencies in a further control population were available from public databases, and these data were combined with the data from the latter group of controls (validation controls). Genotype frequencies were compared between the validation cases and controls. For one region where association was confirmed in this independent cohort, fine-mapping was performed to refine the region of association.

### Patients and controls

All patients with JIA fulfilled the ILAR diagnostic criteria (1), had an age at JIA onset of ≤16 years, were white, and were recruited from across the UK as part of the British Society for Paediatric and Adolescent Rheumatology (BSPAR) National Repository for JIA. Healthy control subjects were identified from blood donor registries and general practitioner records, and samples were obtained. All individuals were recruited with approval of the local ethics committees (North-West Multi-Centre Research Ethics Committee [MREC 99/8/84] and the University of Manchester Committee on the Ethics of Research on Human Beings) and provided informed consent. Genotype data were available from public databases for additional population samples recruited as part of the 1958 birth cohort. All controls were white and were from the UK.

#### Discovery cohort

The 279 JIA cases in the discovery set had a mean age at onset of 4.8 years, and 72% were female. JIA subtypes were as follows: persistent oligoarthritis (n = 133), extended oligoarthritis (n = 80), rheumatoid factor (RF)–negative polyarthritis (n = 57), and RF-positive polyarthritis (n = 9). The controls consisted of 184 subjects with no history of inflammatory arthritis; 48% of them were female.

#### Validation cohort

The 321 JIA cases in the validation set had a mean age at onset of 6.3 years, and 79% were female. JIA subtypes were as follows: persistent oligoarthritis (n = 74), extended oligoarthritis (n = 67), RF-negative polyarthritis (n = 120), and RF-positive polyarthritis (n = 60). Controls comprised 544 individuals with no history of inflammatory arthritis, as well as up to 1,480 individuals from the 1958 UK birth cohort.

#### Fine-mapping cohort

The 654 JIA cases in the fine-mapping cohort had a mean age at onset of 6.9 years, and 68% were female. This cohort represents a combined set of all ILAR JIA subgroups, as follows: persistent oligoarthritis (n = 194), extended oligoarthritis (n = 86), RF-negative polyarthritis (n = 138), RF-positive polyarthritis (n = 35), systemic JIA (n = 115), enthesitis-related JIA (n = 28), psoriatic JIA (n = 51), and unclassified (n = 7). Controls comprised 367 individuals with no history of inflammatory arthritis, as well as up to 1,480 individuals from the 1958 UK birth cohort. This is not an independent data set, and some of the cases and controls in this set were included in the discovery and validation cohorts.

### Genotyping

#### Discovery cohort

The samples in the discovery cohort (n = 463) were processed according to the instructions provided in the Affymetrix GeneChip Human Mapping 100K Assay Manual (online at http://www.affymetrix.com). Samples with a <93% genotype call rate were dropped from the analysis. SNPs that had a minor allele frequency of <5%, that failed to genotype in >5% of samples, or that had a Hardy-Weinberg equilibrium *P* value of <0.0001 in controls were excluded from the analysis.

#### Validation cohort

SNPs showing evidence of an association with JIA in the discovery cohort at *P* ≤ 0.001 were selected for testing in the validation cohort. Samples were genotyped using Sequenom MassArray genotyping technology according to the manufacturer's instructions (Sequenom, San Diego, CA; online at http://www.sequenom.com).

#### Fine-mapping of associated regions

The genotype data from the HapMap project (online at http://www.HapMap.org) in the CEPH population (i.e., Utah residents with ancestry from northern and western Europe; collected by the Centre d'Étude du Polymorphisme Humain [CEPH]) were used to determine pairwise tagging SNPs, using the Tagger software (online at http://www.broad.mit.edu/tools/software.html). The fine-mapping cohort was genotyped using Sequenom MassArray genotyping technology, as in the validation cohort.

### Statistical analysis

Genotype and allele frequencies were compared between cases and controls using Stata version 9 SE (StataCorp, College Station, TX) and Plink (online at http://pngu.mgh.harvard.edu/~purcell/plink/) software. The Armitage test for trend was used to test for association. Stratification by ILAR subtype for associated *VTCN1* SNPs was also performed.

## RESULTS

### Findings in the discovery set

A total of 112,496 SNPs were genotyped in the discovery sample. Since the power calculations for the discovery set had been based upon the detection of modest effect sizes for common alleles, we excluded SNPs with a minor allele frequency of <5% (n = 21,707). A further 1,883 SNPs were excluded because of failure to genotype in >5% of samples, and 224 SNPs were excluded because of significant deviation from Hardy-Weinberg equilibrium. A total of 88,682 autosomal SNPs were therefore analyzed for association with JIA, and 84,235 of these SNPs (95%) had >98% complete genotype data.

One hundred twelve autosomal SNPs were associated with JIA at *P* ≤ 0.001, and these SNPs were selected for genotyping in the validation cohort. (A table of the 112 SNPs taken through for analysis in the validation cohort is available upon request from the corresponding author.) One SNP (rs2187684) was situated in the *HLA* region on chromosome 6p21. Two SNPs that mapped to the *PTPN22* region, which were almost perfectly correlated with each other but which showed only modest correlation with the known *PTPN22* functional variant (rs2476601; r^2^ = 0.27 for rs1217407 and R620W), were weakly associated with JIA (*P* = 0.015 for rs1217407 and *P* = 0.014 for rs1217380).

### Findings in the validation cohort

Of the 112 SNPs associated with JIA, 102 were successfully genotyped in the validation cohort (call rate >90%, Hardy-Weinberg equilibrium at *P* > 0.001). Of these 102 SNPs, 47 had been directly genotyped in the 1958 UK birth cohort controls, whereas imputed data were available for the remaining 55 SNPs (4). Of the 55 imputed SNPs, 48 had confidence scores exceeding 95% (Table 1).

**Table 1:** Single-nucleotide polymorphisms associated with juvenile idiopathic arthritis in the validation cohort*

						Genotype frequency, no. (%)								
				MAF		Cases (n = 321)			Controls (n = 2,024)					
Marker	Chr.	Position	HWE in controls	Cases (n = 321)	Controls (n = 2,024)	11	12	22	11	12	22	Birth cohort directly genotyped (CS)	*P* for trend	OR (95% CI) for allele
rs2187684	6	32872697	0.1	0.28	0.38	12 (6.6)	76 (41.7)	94 (51.6)	297 (15.5)	868 (45.4)	745 (39.0)	No (0.99)	0.00006	0.61 (0.48–0.78)
rs2358820	1	117513434	0.65	0.04	0.08	0 (0)	14 (7.7)	168 (92.3)	14 (0.8)	283 (14.8)	1,612 (84.4)	No (0.99)	0.003	0.45 (0.26–0.78)
rs939898	12	90191601	0.52	0.17	0.21	5 (2.0)	74 (28.9)	177 (69.1)	73 (4.1)	594 (33.8)	1,092 (62.1)	No (0.99)	0.01	0.74 (0.57–0.94)
rs9311745	3	59976865	1	0.05	0.03	1 (0.4)	23 (8.9)	233 (90.7)	1 (0.1)	102 (5.8)	1,655 (94.1)	No (0.97)	0.02	1.68 (1.07–2.62)
rs2833547	21	32171851	0.91	0.23	0.28	12 (6.6)	60 (33.2)	109 (60.2)	148 (8.0)	743 (40.4)	948 (51.6)	Yes (NA)	0.04	0.77 (0.59–0.99)
rs1074044	13	87841327	0.21	0.53	0.48	44 (24.9)	99 (55.9)	34 (19.2)	430 (23.2)	895 (48.4)	525 (28.4)	Yes (NA)	0.05	1.24 (1.0–1.54)

* Chr. = chromosome; HWE = Hardy-Weinberg equilibrium; MAF = minor allele frequency; CS = confidence score; OR = odds ratio; 95% CI = 95% confidence interval; NA = not applicable.

Of the 102 SNPs tested, 11 were associated with JIA at *P* ≤ 0.05, 6 of which showed association with the same allele as at the discovery stage (Tables 1 and 2). The most strongly associated SNP (rs2187684) mapped to the *HLA* region (∼207 kb from the *HLA–DRB1* locus and ∼153 kb from the *HLA–DQB1* locus), while the second strongest association was with a SNP that mapped to the *VTCN1* gene, rs2358820. The *VTCN1* gene was therefore investigated further.

**Table 2:** Comparison of single-nucleotide polymorphisms found to be associated with juvenile idiopathic arthritis in the discovery and validation stages*

		Discovery stage					Validation stage				
			MAF					MAF			
Marker	Chr.	HWE in controls	Cases (n = 279)	Controls (n = 184)	*P* for trend	OR (95% CI) for allele	HWE in controls	Cases (n = 321)	Controls (n = 2,024)	*P* for trend	OR (95% CI) for allele
rs2187684	6	0.09	0.28	0.38	0.0008	0.62 (0.47–0.82)	0.1	0.28	0.38	0.00006	0.61 (0.48–0.78)
rs2358820	1	0.22	0.04	0.1	0.0004	0.4 (0.23–0.68)	0.65	0.04	0.08	0.003	0.45 (0.26–0.78)
rs939898	12	0.05	0.16	0.25	0.0004	0.56 (0.40–0.78)	0.52	0.17	0.21	0.01	0.74 (0.57–0.94)
rs9311745	3	1	0.08	0.02	0.0001	3.67 (1.79–7.5)	1	0.05	0.03	0.02	1.68 (1.07–2.62)
rs2833547	21	0.62	0.22	0.33	0.0006	0.6 (0.44–0.8)	0.91	0.23	0.28	0.04	0.77 (0.59–0.99)
rs1074044	13	0.09	0.56	0.43	0.0001	1.67 (1.27–2.19)	0.21	0.53	0.48	0.05	1.24 (1.0–1.54)

* Chr. = chromosome; HWE = Hardy-Weinberg equilibrium; MAF = minor allele frequency; OR = odds ratio; 95% CI = 95% confidence interval.

### Results of fine-mapping of the *VTCN1* gene

According to the HapMap data, 70 SNPs mapped to the *VTCN1* gene, but only 27 were required to tag the region in order to provide 100% coverage of all nongenotyped SNPs at an r^2^ level of >0.8. Of these 27 SNPs selected for genotyping, assays could not be designed for 2. The 25 remaining SNPs captured 96% of the known variation across the gene. For these 25 SNPs, 7 had been genotyped directly in the 1958 UK birth cohort controls, whereas imputed data were available for the remaining 18 SNPs (4). Of the 18 imputed SNPs, 8 had a confidence score of >95% (Table 3). Ten SNPs mapping to the *VTCN1* gene showed a nominal association with JIA at *P* < 0.05. Seven of them (rs12046117, rs10923223, rs7415876, rs12038533, rs6669320, rs10923217, and rs4376721) lay within intron 1, rs2051047 lay within intron 3, rs2358817 lay within intron 4, and rs6673837 lay in the 3′ region of the gene (Table 3).

**Table 3:** Fine-mapping of single-nucleotide polymorphisms associated with juvenile idiopathic arthritis across the *VTCN1* gene on chromosome 2*

					Genotype frequency, no. (%)								
			MAF		Cases (n = 654)			Controls (n = 1,847)					
Marker	Position	HWE in controls	Cases (n = 654)	Controls (n = 1,847)	11	12	22	11	12	22	Birth cohort directly genotyped (CS)	*P* for trend	OR (95% CI) for allele
rs7546885	117482038	0.5098	0.39	0.39	92 (15.9)	272 (46.9)	216 (37.2)	284 (15.4)	877 (47.6)	683 (37.0)	No (0.97)	0.94	1.0 (0.88–1.15)
rs6673837†	117487515	0.2352	0.22	0.20	22 (3.8)	214 (37.0)	343 (59.2)	66 (3.6)	595 (32.2)	1,186 (64.2)	Yes	0.05	1.16 (1.0–1.37)
rs1937956	117488648	0.3758	0.28	0.28	46 (7.9)	236 (40.7)	298 (51.4)	146 (7.9)	755 (40.9)	946 (51.2)	Yes	0.96	1.0 (0.86–1.15)
rs7523182	117489576	0.1902	0.13	0.12	10 (1.7)	136 (23.4)	434 (74.8)	26 (1.4)	395 (21.4)	1,424 (77.2)	No (0.91)	0.23	1.13 (0.93–1.37)
rs2358817†	117492281	1	0.06	0.09	5 (0.9)	59 (10.2)	513 (88.9)	13 (0.7)	290 (15.7)	1,544 (83.6)	Yes	0.005	0.68 (0.52–0.89)
rs2051047†	117497560	1	0.06	0.08	5 (0.9)	61 (10.5)	514 (88.6)	13 (0.7)	286 (15.5)	1,547 (83.8)	Yes	0.01	0.71 (0.54–0.92)
rs4659159	117517799	0.1918	0.36	0.36	79 (13.8)	257 (44.9)	236 (41.3)	246 (13.4)	842 (45.9)	748 (40.7)	No (0.98)	0.97	1.0 (0.87–1.15)
rs12739378	117522103	0.1187	0.01	0.02	0 (0)	17 (2.9)	563 (97.1)	1 (0.1)	58 (3.1)	1,786 (96.8)	No (0.99)	0.70	9.0 (0.52–1.55)
rs4376721†	117523703	0.8883	0.31	0.28	58 (10.0)	245 (42.2)	277 (47.8)	142 (7.8)	713 (40.1)	925 (52.1)	Yes	0.04	1.16 (1.0–1.34)
rs2185815	117525154	0.2813	0.22	0.20	31 (5.3)	196 (33.8)	353 (60.9)	73 (4.1)	579 (32.2)	1,145 (63.7)	Yes	0.13	1.13 (0.96–1.33)
rs10923217†	117531571	0.9169	0.49	0.45	137 (23.7)	289 (49.9)	153 (26.4)	365 (19.8)	919 (49.9)	559 (30.3)	No (0.77)	0.02	1.17 (1.02–1.33)
rs12028520	117531746	0.6048	0.10	0.10	8 (1.4)	103 (18.0)	461 (80.6)	20 (1.1)	327 (17.9)	1,483 (81.0)	No (0.87)	0.71	1.04 (0.84–1.29)
rs6669320†	117532146	0.5172	0.14	0.17	8 (1.4)	151 (26.0)	421 (72.6)	53 (2.9)	531 (28.8)	1,259 (68.3)	No (0.84)	0.02	0.8 (0.67–0.97)
rs12030415	117533495	0.3132	0.28	0.30	42 (7.3)	235 (40.6)	302 (52.2)	160 (8.7)	801 (43.5)	882 (47.9)	No (0.74)	0.06	0.87 (0.75–1.0)
rs12038533†	117534039	0.02835	0.08	0.10	8 (1.4)	77 (13.3)	493 (85.3)	28 (1.6)	319 (17.8)	1,441 (80.6)	Yes	0.02	0.75 (0.59–0.95)
rs7415876†	117547679	0.4431	0.17	0.20	14 (2.4)	171 (29.5)	395 (68.1)	70 (3.8)	592 (32.1)	1,181 (64.1)	No (0.84)	0.04	0.84 (0.7–0.99)
rs10801937	117547734	0.5531	0.33	0.31	59 (10.2)	267 (46.0)	254 (43.8)	171 (9.3)	818 (44.4)	854 (46.3)	No (0.77)	0.26	1.08 (0.94–1.25)
rs10923223†	117548096	0.03158	0.16	0.12	17 (3.0)	148 (25.8)	409 (71.3)	27 (1.5)	370 (20.1)	1,446 (78.5)	No (0.90)	0.0001	1.45 (1.2–1.75)
rs12076501	117548162	0.2612	0.30	0.28	45 (8.0)	247 (43.9)	271 (48.1)	154 (8.4)	740 (40.2)	949 (51.5)	No (0.82)	0.33	1.07 (0.93–1.24)
rs6674646	117551332	0.2769	0.02	0.03	1 (0.2)	24 (4.1)	555 (95.7)	1 (0.1)	96 (5.2)	1,749 (94.7)	No (0.99)	0.44	0.84 (0.54–1.3)
rs12046117†	117552888	0.0993	0.13	0.09	11 (1.9)	133 (23.0)	435 (75.1)	17 (0.9)	294 (15.9)	1,534 (83.1)	No (0.90)	1 × 10^−6^	1.58 (1.29–1.94)
rs10158166	117555530	0.05257	0.13	0.14	12 (2.1)	129 (22.2)	439 (75.7)	37 (2.0)	426 (23.1)	1,382 (74.9)	No (0.96)	0.75	0.97 (0.8–1.18)
rs4659229	117557323	0.07875	0.10	0.12	8 (1.4)	100 (17.2)	472 (81.4)	25 (1.4)	377 (20.4)	1,443 (78.2)	No (0.99)	0.14	0.85 (0.68–1.05)
rs7553082	117557349	1	0.01	0.01	0 (0)	17 (2.9)	563 (97.1)	0 (0)	51 (2.8)	1,795 (97.2)	No (0.99)	0.83	1.06 (0.61–1.85)
rs6690137	117557755	0.09613	0.12	0.13	7 (1.2)	120 (20.8)	451 (78.0)	33 (1.8)	414 (22.4)	1,398 (75.8)	No (0.99)	0.21	0.88 (0.71–1.07)

* HWE = Hardy-Weinberg equilibrium; MAF = minor allele frequency; CS = confidence score; OR = odds ratio; 95% CI = 95% confidence interval.

† Marker was found to be associated with juvenile idiopathic arthritis at *P* < 0.05.

There was only moderate linkage disequilibrium across the gene as a whole, but the associated SNPs appeared to map to 3 regions within the gene. The SNPs showing the strongest association (rs10923223 and rs12046117) are situated in intron 1 and demonstrated moderate linkage disequilibrium with each other (r^2^ = 0.68). However, this finding should be viewed with caution, since the confidence score for the imputed genotypes in the control population was <95%, suggesting that this may have introduced some bias.

A second group of SNPs in intron 1 showed modest evidence of association, in which the major allele was associated with disease. SNPs rs2358817 and rs2051047 showed stronger evidence of association and mapped to introns 3 and 4, respectively. They demonstrated a strong correlation with each other and probably represent a single effect (r^2^ = 0.83).

This analysis was performed in all JIA subtypes. Reanalysis using only the JIA subtypes included in the original discovery stage did not alter the results. Comparison of genotype counts for *VTCN1* SNPs across the different ILAR subtypes showed no significant differences. (A table showing the ILAR stratification analysis of *VTCN1*-associated SNPs is available upon request from the corresponding author.)

## DISCUSSION

In this study, we used a whole-genome association approach to identify novel JIA susceptibility loci, and for one of these regions, we performed fine-mapping analysis to refine the extent of association. We identified associations between JIA and polymorphisms mapping to the *VTCN1* gene.

The possibility that this finding could represent a false-positive result requires consideration. This could arise as a result of population stratification. However, the Wellcome Trust Case Control Consortium study previously established that there was very little evidence of this across the UK (3). They found only 13 loci exhibiting significant geographic variation, none of which overlapped with loci in the present investigation.

We used a modest threshold for significance (*P* < 0.001), accepting that the majority of SNPs showing association would be false-positives and would therefore fail to be replicated in independent cohorts. We performed no corrections for multiple testing, but instead, took the approach of validation of any significant results in independent cohorts. Using this approach, strong evidence for an association with *HLA* was detected, serving as a proof-of-concept that established JIA susceptibility factors could be identified.

In contrast, SNPs mapping to the other confirmed JIA susceptibility locus, the *PTPN22* gene, showed only weak associations. This is likely due to the low correlation between SNPs on the Affymetrix GeneChip 100K array and the known *PTPN22* causal variant, rs2476601 (r^2^ = 0.27). Furthermore, the Affymetrix 100K GeneChip Array is only estimated to capture ∼35% of known variation across the genome, and it is therefore likely that a number of JIA susceptibility loci remain to be identified. Nonetheless, there have been notable successes in identifying genes that underlie complex diseases using a staged approach in modest sample sizes and with the same SNP density as in this study. Examples include the complement factor H gene, which has been widely confirmed as a susceptibility gene for age-related macular degeneration (5), and a region on chromosome 6q23 (6), which has been confirmed as a rheumatoid arthritis susceptibility locus (7).

It is likely that other JIA-associated genes are present but were not identified in our study because the small sample size used in the discovery set limited the power to detect causal variants with small effect sizes (false-negative); nonetheless, this is the most comprehensive genome-wide association study of JIA to date. The sample size used in the discovery cohort was selected based on its power to detect effect sizes equivalent to the confirmed JIA susceptibility causal variant within the *PTPN22* gene, but we attempted to increase the power by selecting more homogeneous JIA cases, excluding systemic-onset JIA, psoriatic JIA, and enthesitis-related JIA cases. However, the male to female ratio between the case and control groups was not matched in this study, and as such, the observed data are not a true representation of all genetic association.

Despite these limitations, association of the *VTCN1* gene with JIA was detected and replicated. The gene is an interesting candidate for this autoimmune disease, since it is part of a recently identified inhibitory pathway that is important in the prevention of detrimental inflammatory responses, such as the autoimmune response seen in the joints of children with JIA. *VTCN1*, which is also known as B7-H4, is expressed on activated T cells, B cells, monocytes, and dendritic cells (8), and evidence suggests that it plays a role in the negative regulation of T cell responses. It is a member of the B7 family of costimulatory molecules, and it has been proposed that the ligand for *VTCN1* is the B and T lymphocyte attenuator, an inhibitory receptor on T cells. Therefore, it may be involved in the attenuation of inflammatory responses in peripheral tissue (8). All 10 associated variants are intronic, and none has a recognized function as yet. Resequencing and further genotyping in the region will be required to identify the causal variants before functional studies can be undertaken to determine how they predispose to disease.

In summary, we identified 6 regions where there is replicated evidence of association with susceptibility to JIA, including the *VTCN1* gene. Although confirmation of this association in different populations will be important, the identification of *VTCN1* as a JIA susceptibility locus represents a novel pathway by which to explore more about the cause of the disease. In turn, this may lead to novel therapies for this disabling, chronic arthritis of childhood.

## AUTHOR CONTRIBUTIONS

Dr. Hinks had full access to all of the data in the study and takes responsibility for the integrity of the data and the accuracy of the data analysis.

**Study design.** Hinks, Barton, Kennedy, John, Worthington, Thomson.

**Acquisition of data.** Hinks, Eyre, Bowes, Cargill, Wang, Kennedy, Thomson.

**Analysis and interpretation of data.** Hinks, Barton, Shephard, Eyre, Cargill, Wang, Kennedy, John, Worthington, Thomson.

**Manuscript preparation.** Hinks, Barton, Ke, Kennedy, John, Thomson.

**Statistical analysis.** Hinks, Shephard, Cargill, Ke, John.
